# Systematic review of longitudinal studies on daily health behavior and activity of daily living among older adults

**DOI:** 10.3389/fpubh.2025.1419279

**Published:** 2025-02-19

**Authors:** Ling-ying Wang, Zi-yi Hu, Hong-xiu Chen, Hong Zhu, Chun-fen Zhou, Rui-xue Zhang, Meng-lin Tang, Xiu-ying Hu

**Affiliations:** ^1^Critical Care Medicine Department, West China Hospital, Sichuan University/West China School of Nursing, Sichuan University, Chengdu, China; ^2^Innovation Center of Nursing Research and Nursing Key Laboratory of Sichuan Province, West China Hospital, Sichuan University/West China School of Nursing, Sichuan University, Chengdu, China; ^3^Nursing Department, West China Hospital, Sichuan University/West China School of Nursing, Sichuan University, Chengdu, China; ^4^Mental Health Center, West China Hospital, Sichuan University/West China School of Nursing, Sichuan University, Chengdu, China

**Keywords:** daily health behavior, activities of daily living, older adults, healthy life expectancy, longitudinal

## Abstract

**Background:**

Health behavior, as an intervention led by nursing staff, plays a pivotal role in enhancing the health of older adults. However, existing evidence has predominantly focused on isolated aspects (e.g., smoking, alcohol consumption, diet, or exercise), with most studies being cross-sectional, thereby limiting the ability to establish causal relationships between these factors and Activities of Daily Living (ADLs). A comprehensive synthesis of longitudinal studies is required to elucidate the impact of daily health behaviors on ADLs in older adults.

**Objectives:**

Our goal was to assess the predictive relationship between daily health behaviors and ADLs, aiming to better understand their longitudinal interplay among the older adults population.

**Design:**

This systematic review was meticulously conducted following the guidelines of the Preferred Reporting Items for Systematic Reviews and Meta-Analyses (PRISMA) and the recommendations of the Cochrane Community.

**Data sources:**

A thorough search was conducted using Medical Subject Headings (MeSH) terms and associated keywords in databases such as PubMed, Web of Science, Embase, China National Knowledge Infrastructure (CNKI), Chinese Technical Periodicals (VIP), and Wanfang, up to December 2024.

**Methods:**

Two researchers independently screened the articles, and data extraction and verification were carried out for those meeting the inclusion criteria. This review systematically evaluated longitudinal studies examining the correlation between various daily health behaviors and ADL performance in older adults.

**Results:**

13 longitudinal studies were included after excluding duplicates and irrelevant literature. Of these, 12 (92.3%) were deemed high quality, and 1 (7.7%) was considered low quality. Daily exercise was identified as the most influential behavior for preventing ADL disability, with adherence to a Mediterranean diet (MeDi) and moderate alcohol consumption also demonstrating protective effects. In contrast, physical inactivity, a sedentary lifestyle, and smoking were strongly linked to ADL impairment, followed by sleep duration issues, infrequent consumption of fruits and vegetables, lower frequency of soy product intake, and higher energy intake.

**Conclusion:**

Longitudinal data and theoretical literature indicate that daily health behavior indicators predict independence in older adults. However, these findings should be interpreted with caution due to the inability to pool data from the included studies, which exhibited significant variations in the operationalization of the indicators and ADLs. The results highlight the clinical significance of advocating for healthy behaviors to prevent early ADL decline in older adults. Implementing these insights could lead to a substantial reduction in healthcare burdens and an extension of healthy life expectancy.

**Systematic Review Registration:**

https://www.crd.york.ac.uk/prospero/display_record.php?ID=CRD42023491550, identifier CRD42023491550.

## 1 Introduction

Aging refers to the process of growing older, characterized by a series of gradual changes that unfold over time, including physical, mental, and social transformations (1). The population of adults ages 60 years old is estimated to double to 2.1 billion, and 80 years and older is expected to triple to 426 million by 2050 (2). The global trend of an aging population poses many challenges, such as the rising prevalence of chronic diseases, increased financial strains, and labor shortages, thereby amplifying public health concerns and emerging as a formidable challenge to public health systems worldwide (3). Health is as a measure of individual’s ability to achieve their aspirations and satisfy their needs, rather than simply as the absence of disease. Over the past half-century, successful aging has emerged as a central focus of research within the field of gerontology (4, 5).

Preserving the ability to maintain physical and cognitive independence is crucial for a healthy life expectancy in the senior years and is a critical component of successful aging (6). Functional ability refers to individuals’ capacity to engage in daily life and social activities based on their intentions and preferences (2). According to the World Health Organization (7), disability is a “general term for impairments, activity limitations, and participation restrictions, reflecting the negative aspects of the interaction between health conditions, personal factors, and the environment.” The activities of daily living (ADL) and instrumental activities of daily living (IADL) functions are important to older adults (8, 9). ADLs encompass the fundamental activities necessary for self-care, including feeding, transferring, grooming, toileting, bathing, walking, climbing stairs, dressing and undressing, and managing bowel and bladder functions (10, 11). The IADL refers to activities to support daily life within the home and community that often require more complex interactions than those used in ADLs. Examples of such activities include financial management, housekeeping, shopping for groceries, making telephone calls, and taking medication (8).

Advances in medical technology and practice, coupled with improvements in social and public health, have significantly increased human life expectancy. However, these additional years of life may not necessarily be accompanied by good physical health, cognitive functioning, or psychosocial well-being. Throughout the lifespan, the individual accumulation of various risk and protective factors can lead to substantial differences in the levels and trajectories of aging (12). Promoting physiological and psychosocial well-being across the lifespan carries important health, policy, and economic implications, particularly in the context of the global demographic shift (13). In addition to investigating specific disorders and the negative aspects of aging, research into the factors that contribute to successful aging can provide valuable insights into how the later years of life might be enhanced.

Health behavior, as an intervention that can be facilitated by nursing staff, plays a significant role in enhancing the health of older adults individuals. It encompasses proactive measures taken by seniors to prevent illness and sustain well-being, which include modifying risky lifestyles, mitigating or eliminating health-hazardous behaviors like smoking and excessive alcohol consumption, and adopting healthy practices such as regular physical activity and routine medical check-ups, as well as following medical advice. Several studies have identified key factors that promote successful aging, including a healthy diet with moderate food intake, regular physical activity, and an active social lifestyle (14), as well as favorable environmental conditions (15). For example, populations in the so-called “blue zones”—regions such as Okinawa, Sardinia, and Costa Rica—are known for their high proportion of individuals living well into old age (15–17). A 20-year follow-up cohort study, which examined individuals aged 70 and older, explored the likelihood of reaching the age of 90 (18). The findings revealed gender differences in the factors influencing longevity: men who were physically active had a higher chance of survival, while women who were physically active, relatively healthy, and satisfied with their income and housing arrangements were more likely to become nonagenarians (18). At the same time, adherence to positive health behaviors is essential in managing noncommunicable diseases and substantially curtailing healthcare costs within the aging demographic (19, 20).

Previous research (21–23) has concentrated mainly on individual aspects (e.g., smoking, alcohol consumption, diet, or exercise), with the majority being cross-sectional studies. This approach restricts the capacity to identify potential causal connections between these behaviors and ADLs. A previous longitudinal study investigated how the emotional dynamics of marital relationships influence subsequent health outcomes through behavioral mechanisms. It found that diet and exercise serve as key mechanisms linking marital dysfunction to health over a 20-year period (24). Longitudinal studies, through long-term tracking and multiple measurements, can deeply reveal the progression of loss of ADL and the long-term impact of health behaviors on functional status.

Gaining insight into the predictive power of daily health behaviors on ADL disability is of clinical importance, providing a foundation for targeted interventions aimed at preserving and enhancing the ADL of older adults. To bridge this gap in knowledge, our systematic review consolidates evidence from longitudinal studies to elucidate the interplay between daily health behaviors and ADLs among individuals aged 60 and older.

## 2 Methods

### 2.1 Data sources and search strategy

This systematic review was meticulously designed and executed in strict compliance with the Preferred Reporting Items for Systematic Reviews and Meta-Analyses (PRISMA) (25) and the guidelines provided by the Cochrane Community (26), ensuring methodological rigor in planning, conducting, and reporting our research. Our literature search extended to December 2024 and encompassed PubMed, Web of Science, Embase, China National Knowledge Infrastructure (CNKI), Chinese Technical Periodicals (VIP), and Wanfang databases. Our review is registered with PROSPERO under the number CRD42023491550.

To comprehensively capture a wide array of daily health behaviors that influence ADLs in older adults, we employed a strategic blend of Medical Subject Headings (MeSH) and free-text terms. The MeSH terms we utilized included “activities of daily living” and “aged.” The following search strategies were employed to navigate PubMed: (“activities of daily living” [MeSH terms] OR “activities of daily living” [all fields] OR “ability of daily living” [all fields] OR “daily living” [all fields] OR “disable” [all fields]) AND (“health behavior” [all fields] OR “behavior” [all fields] OR “physical activity” [all fields] OR “physical exercise” [all fields] OR “acute exercise” [all fields] OR “isometric exercise” [all fields] OR “exercise training” [all fields] OR “gym” [all fields] OR “ambulation” [all fields] OR “cycling” [all fields] OR “diet” [all fields] OR “dietary habits” [all fields] OR “difficulty falling asleep” [all fields] OR “wake up early” [all fields] OR “sleep habits” [all fields] OR “sleep duration” [all fields] OR “tobacco” [all fields] OR “alcohol consumption” [all fields] OR “alcohol withdrawal” [all fields]) AND (“aged” [MeSH terms] OR “older people” [all fields]).

Our systematic review employed a consistent search methodology across various electronic databases, utilizing MeSH and key free-text terms. In cases where data was incomplete, we took the initiative to contact the studies’ authors to seek out additional information, ensuring that our analysis was as comprehensive and meticulous as possible. Furthermore, we meticulously reviewed the references cited within the selected studies to identify any pertinent articles that may have been overlooked.

### 2.2 Inclusion and exclusion criteria

We established explicit inclusion and exclusion criteria before initiating the study. The criteria for inclusion were as follows:

- The study must provide data on the functional independence of individuals aged 60 years or older concerning their ADLs.
- It must have measured critical potential confounding variables, such as baseline ADL independence, age, sex, functional capacity, current health status, etc., and statistically adjusted for the impact of these variables on the relationship between the exposure and outcomes.
- The study must present longitudinal data featuring at least two comparable sets of ADL status measurements to enable the analysis to infer causality between health behaviors and changes in ADL status over time.

The exclusion criteria were defined as:

- Studies involving participants younger than 60 were excluded to concentrate solely on the older adults population.
- We omitted studies that were not longitudinal to preserve a uniform methodological framework.
- Studies that did not report effect sizes or provide bidirectional data, or those from which authors did not furnish additional required information upon request, were also excluded.

### 2.3 Selection of studies and data collection

All English and Chinese publications from the databases’ inception up to December, 2024, were considered for inclusion if they examined the relationship between daily health behaviors and disability in ADL among older adults aged 60 years and above. This encompassed diverse populations, including community-dwelling, institutionalized, hospitalized, rural, and urban individuals.

During the initial selection phase, three researchers (LY, CF and RX) independently assessed the titles and abstracts against the inclusion criteria, identifying potentially relevant papers. Any disagreements were addressed through discussion, leading to a consensus. The search results were further enhanced by scrutinizing the references cited in critical papers.

In the subsequent selection round, three researchers (LY, ZY and HZ) independently retrieved the full texts of each paper deemed potentially eligible and assessed their suitability based on the inclusion criteria. Any opinions divergences were deliberated with a third researcher (XY) until a unanimous agreement was reached. Ultimately, data from the selected longitudinal studies were systematically extracted and organized into an Excel spreadsheet. This compilation included details such as the study title, authors, publication year and journal, the country of the study’s execution, demographic information about the study population (age, size, setting), the methodology for ADL assessment, follow-up duration, study outcomes, statistical approaches, and findings.

### 2.4 Review of study strength and quality

To rigorously assess the methodological quality of the included studies, we employed the Newcastle-Ottawa Scale (NOS). The initial quality assessment was performed by one researcher (LY), followed by an independent verification by a second reviewer (CF) to ensure consistency and precision in the evaluation process.

The NOS is recognized for its holistic approach, encompassing eight criteria across three distinct domains: selection, comparability, and outcome or exposure. These criteria are specifically tailored to accommodate the study’s design, whether it be a cohort or case–control study. This framework facilitates a detailed and nuanced assessment of the quality of each study. For each criterion, a set of response options is provided, allowing for a semi-quantitative evaluation of study quality. The NOS employs a star system, where the highest quality studies can be awarded up to one star for each criterion, except the comparability domain, which permits the allocation of two stars. Consequently, the overall score on the NOS scale ranges from zero to nine stars, providing a clear and quantifiable measure of study quality (27).

### 2.5 Data extraction

Considering the significant heterogeneity in the measurement methods for health behavior indicators and ADL disability across the studies, we decided against pooling data for meta-analysis. This decision was made to ensure that the diversity in study design and population characteristics would be consistent with the unique findings of each study.

To evaluate the predictive power of each health behavior indicator on ADL disability, we conducted a qualitative synthesis by counting the number of studies that reported a statistically significant increase in risk. We then categorized these studies into two groups: those that exclusively included participants who were not disabled at baseline and those that included a mix of participants, both with and without disability at baseline. Studies with participants free of disability at baseline were given greater weight (++), reflecting the more substantial evidence for a predictive relationship. In contrast, studies that included both disabled and non-disabled participants at baseline were given a single weight (+). For studies that did not reveal a statistically significant predictive link between health behavior indicators and ADL disability, we assigned a negative weight (−). This acknowledges their valuable contribution to the body of evidence while indicating the absence of a predictive association (28).

## 3 Results

### 3.1 Study selection and characteristics

**Figure 1:**
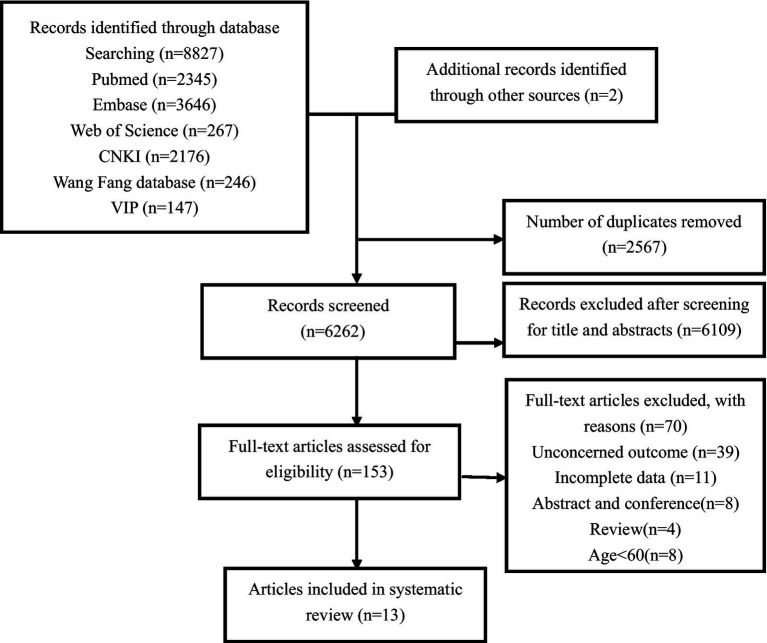
Flowchart of the selection of studies for inclusion in the systematic review.

Our extensive search strategy yielded an initial pool of 8,827 studies. After rigorously eliminating duplicates, we carefully examined 6,262 titles and abstracts, leading to the full-text assessment of 153 studies for their eligibility. The meticulous selection process concluded with including 13 longitudinal studies (as depicted in Figure 1). These studies featured a diverse geographic spread, with the first authors hailing from Italy, Sweden, Norway, the United States, China, Brazil, Japan, and France. These studies involved a substantial cohort of 29,180 participants, achieving a balanced gender distribution with 13,990 males and 15,190 females (29–41).

Table 1 provides a chronological summary of the included studies, categorized by their publication year. Table 2, on the other hand, details the principal findings from these studies, shedding light on the extensive scope of research undertaken.

**Table 1:** Characteristics of the participants and outcome measures of the studies included in the systematic review.

Author/year	Country	Cohort	Sample Size	Age, in years	Female, *n* (%)	Activities of daily living: outcome measure	Follow-up
Zhou/2024 (29)	China	CLHLS	2,471	78.10 ± 8.63	1,157 (46.8)	Katz	6-year
Zhu/2024 (30)	China	Community-dwelling	5,154	70.86 ± 6.23	2,979 (57.8)	Assessment Form for the Self-care Ability of the Elderly	Median time: 3.30 years
Kojima/2023 (31)	Japan	Community-dwelling women	1,114	78.4 ± 2.7	1,114 (100)	Katz	4-year
Liu Y/2021 (32)	China	CFPS	750	≥60	368 (49.1)	The Physical Activity Scale	2-year
Heiland/2019 (33)	Sweden	SNAC-K	1756	70.6 ± 9.6	1,154 (65.7)	Bathing, dressing, toileting, transferring, and eating	9-year, (6.3 ± 1.7)
Jonkman/2018 (34)	Italy, Holand	InCHIANTI, LASA	798	Male: 67.4 ± 2.1 Female: 67.5 ± 2.1	429 (53.8)	Dressing and undressing, Sitting down and standing up	9-year
Storeng/2018 (35)	Norway	The HUNT Study	5,050	60–69	2,738 (5432)	Needing assistance in one or more basic reported in HUNT3	12-year
Artaud/2013 (36)	France	Dijon center of Three-City stud	3,982	73.9 ± 5.2	861 (69.7)	Katz	12-year
Alexandre/2012 (37)	Brazil	The SABE study	1,634	Male: 68.3 ± 0.4 Female: 68.8 ± 0.3	933 (57.1)	Kate	6-year
Feart/2011 (38)	France	The Three-City (3C) study	1,410	≥65	883 (62.6)	Katz, Lawton–Brody scale	7-year
Balzi/2010 (39)	Italy	The InCHIANTI study	897	≥65	409 (47.8)	/	3-year
Wang/2002 (40)	America	The Adult Changes in Thought (ACT) Study	2,581	≥65	1,520 (58.9)	Walking around a house, bathing or taking shower, dressing themselves, getting out of a bed or chair, feeding themselves, and using a toilet.	3.4 year (range 0–7 years)
Wu/1999 (41)	China	In the four districts of the Taipei metropolitan area	1,583	≥65	645 (40.7)	The six ADL-related items	3-year

CLHLS, Chinese Longitudinal Healthy Longevity Survey; Katz, modified Katz Index; CFPS, China Family Panel Studies; SNAC-K, the Swedish National Study on Aging and Care in Kungsholmen; InCHIANTI, Chianti Study; LASA, the Longitudinal Aging Study Amsterdam; HUNT, The Nord-Trøndelag Health Study; SABE, a multicenter survey carried out in the main urban centers of seven countries in Latin America and the Caribbean.

**Table 2:** Results of the studies included in the systematic review.

Author/year	Factors	Definition/classification of health behaviors	Key findings
Zhou/2024 (29)	Non-exercise physical activity (NEPA)	NEPA: including keeping domestic animals or pets, gardening and housework.	Protective Factors: NEPA
Zhu/2024 (30)	Frequency of physical exercise	Physical exercise: categorized: never exercise, occasionally exercise and every day exercise.	Protective Factors: frequency of physical exercise.
Kojima/2023 (31)	Soy product intake frequency	Soy product intake frequency: categorized: almost every day, once every 2 days, once or twice a week and almost never.	Protective Factors: Soy product intake frequency
Liu Y/2021 (32)	Age, education level, household registration, employment status, the annual total family expenditure and the annual total family income in 2015, whether the family is in debt, whether they (or their spouse) own a house, exercise time, middle and high intensity leisure activities, mental leisure activities, low intensity leisure activities, cardiovascular diseases and malignant respiratory diseases	Middle and high intensity leisure activities: including ball games, dancing, and traveling, etc. Mental leisure activities: including playing chess, surfing the internet, etc. Low intensity leisure activities: including walking, gardening, and keeping pets, etc.	Protective factors: middle and high intensity leisure activities; low intensity leisure activities; mental leisure activities Risk Factors: exercise time
Heiland/2019 (33)	Cardiovascular risk factors (physical inactivity, alcohol consumption, smoking, high blood pressure, diabetes, high body mass index, high levels of total cholesterol, high C-reactive protein) and walking speed	Physical activity was assessed through self-administered questionnaires on the frequency of light physical activity (eg, walks on the street and parks, short bike rides, light gymnastics, golf) and high intensity physical activity (eg, jogging, brisk long walks, heavy garden work, long bike rides, intensive gymnastics, long-distance skating, skiing, swimming, ball sports) in the last 12 months. This question was then dichotomized into active (weekly participation of moderate-to-vigorous intensity) and inactive (less than weekly participation of moderate-to-vigorous intensity).	Risk factors: physical inactivity
Jonkman/2018 (34)	Male: gait speed, fear of falling and alcohol intake Female: gait speed, age, living alone, economic satisfaction, balance, physical activity, BMI, and cardiovascular disease	Alcohol intake: number of alcoholic glasses per week, categorized: None; ≥1 glasses Physical activity: InCHIANTI Weekly intensity categorized: Hardly any physical activity (low); Mostly sitting/some walking (low); Light exercise 2–4 h/week (moderate); Moderate 1–2 h or light >4 h/wk. (moderate); Moderate exercise >3 h/wk.; Intense exercise many times/wk. InCHIANTI participants were categorized in three levels: high level (intense/moderate exercise>3 h/wk); moderate level (light/moderate exercise 1–2 h/wk); low level (hardly any activity/some walking). LASA: LAPAQ questionnaire for measuring time spent last two weeks on bicycling, walking outdoors, and doing sports. LASA participants were categorized based on sex-specific tertiles: high level (highest tertile); moderate level (medium tertile); low level (lowest tertile).	Protective factors: alcohol intake (male, severe subgroup); moderate physical activity levels (female, intermediate and severe subgroups) Risk factors: alcohol intake (intermediate subgroup)
Storeng/2018 (35)	Lifestyle risk factors (smoking, alcohol, physical inactivity, sitting time, sleep and social participation), depression, anxiety, self-rated health and life satisfaction	Smoking: being a daily smoker Alcohol: scoring 2 or more (out of 4) on the Cut down, Annoyed, Guilty and Eye opener (CAGE) questionnaire for problematic drinking behavior. Physical inactivity: participating only a few times a year or never in social activities Sitting time: sitting 8 h or more daily Sleep: sleeping 6 h or less or 10 h or more Social participation: less than 3 h of light physical activity and no hard physical activity a week	Risk factors: excessive sitting time, short or prolonged sleeping time, and physical inactivity
Artaud/2013 (36)	Unhealthy behaviors (low/intermediate physical activity, consuming fruit and vegetables less than once a day, current smoking/short term ex-smoking, never/former/heavy alcohol drinking)	Physical activity: assessed through questions on frequency of daily walking and exercise (for example, gym, swimming, cycling) and categorized as high (walking more than one hour a day and exercising more than once a week), low (walking less than one hour a day and exercising less than once a week), and intermediate (all others). Consumption of fruit and vegetables: responses were on a six point scale (“never” to “at least once a day”). Smoking status: categorized: never smoker, long term ex-smoker (quit smoking at least 15 years before baseline), short term ex-smoker (quit smoking less than 15 years before baseline), and current smoker. Consumption of alcohol: categorized: never drinker, former drinker, light to moderate drinker (1–21 alcoholic drinks a week for men and 1–14 for women), and heavy drinker.	Risk factors: low/intermediate physical activity; consuming fruit and vegetables less than once a day; current smoking/short term ex-smoking
Alexandre/2012 (37)	Age, depressive symptoms, stroke, slowness on the sit-and-stand test, osteoarthritis, sedentary lifestyle, cognitive performance and handgrip strength	Physical activity: Individuals were considered active when reporting physical activity at least three times a week over the previous 12 months. The opposite is a sedentary lifestyle.	Risk factors: sedentary lifestyle (female)
Feart/2011 (38)	Mediterranean-type diet (MeDi)	The MeDi score was generated by adding the scores for each food category for each participant. Thus, the MeDi score could range from 0 to 9, with higher scores indicating greater MeDi adherence. Three MeDi categories (Low MeDi adherence, score 0–3; Middle MeDi adherence, score 4–5; or High MeDi adherence, score 6–9) were defined.	Protective factors: MeDi adherence
Balzi/2010 (39)	Hypertension, average daily intakes of energy (kcal), physical activity	Physical activity level in the previous year was considered as an ordinal variable and scored into seven progressive grades, from 0 (hardly any physical activity) up to 7 (intense exercise many times/week) by using a modified version of a standard questionnaire. Physical activity was dichotomized (absent–light vs. moderate). Average daily intakes of energy (kcal) and alcohol were estimated using the European Prospective Investigation into Cancer and Nutrition food frequency questionnaire.	Protective factors: physical activity Risk factors: energy intake
Wang/2002 (40)	Medical Conditions (diabetes mellitus, hypertension, coronary heart disease, cerebrovascular dis ease (CVD), osteoporosis, arthritis, and cancer), low cognitive function, depression, smoking, exercise and moderate alcohol use	Alcohol use: nondrinkers (<5 drinks/year); Drinkers (≥5 drinks/year without problem); Problem drinkers (who reported any of the alcohol-related problem). This so-called moderate pattern of alcohol use were participants consumed five drinks or more a year and did not report any alcohol-related problem. Smoking: categorized: smoked ≥100 cigarettes; current smoker. Physical activities: assessed at baseline and follow-up by asking subjects the number of days per week they did each of the following activities at least 15 min at a time: walking for exercise, hiking, bicycling or stationary bicycle, aerobics or calisthenics, swimming, water aerobics, weight training or strengthening, or other exercise. Subjects who participated in any of these forms of exercise at least three times per week were classified as performing regular exercise.	Protective factors: exercise and moderate alcohol use Risk factors: smoking
Wu/1999 (41)	Age, exercises (folk dancing, hiking, jogging, or walking)	Exercises: any one of the analyzed (folk dancing, hiking, jogging, or walking) at least twice a week were considered as exercisers.	Protective factors: routine exercise

BMI, body mass index; InCHIANTI, Chianti Study; LASA, the Longitudinal Aging Study Amsterdam.

The study designs exhibited considerable diversity, with sample sizes varying from 750 to 5,050 participants. Significantly, 12 studies performed retrospective cohort analyses (29, 30, 32–41), while one adopted a prospective methodology (31). The interpretation of ADLs differed among the studies as well. Some defined disability as reliance on assistance for ADLs at the follow-up, others as experiencing difficulty with ADLs at follow-up, and some considered it as an outcome measure.

The duration of the follow-up periods extended from 2 to 12 years, underscoring the long-term commitment of these studies. An overwhelming majority (84.6%) of the studies were published within 14 years from 2010 to 2024. Each study employed stringent multivariate analysis techniques to ensure that potential confounding variables were effectively controlled for, thus bolstering the credibility and robustness of the findings.

### 3.2 Quality of studies

We utilized the Newcastle-Ottawa Scale (NOS) to rate and categorize the quality of the studies, as depicted in Table 3. The quality scores of the 13 included studies ranged from 4 to 6 stars. Based on the NOS criteria, 12 studies (92.3%) were deemed high quality, while 1 study (7.7%) was identified as low quality. The aspects where compliance was less frequently observed related to the absence of certain elements at the commencement of the study, during the outcome assessment phase, or throughout the follow-up period.

**Table 3:** Newcastle–Ottawa scale (NOS) score of studies included in the systematic review.

Year	Selection			Comparability		Outcome			Total
	Representatives	Selection exposed cohort	Ascertainment	Result not present at start of the study	Comparability for confounders	Assessment of outcome	Follow-up duration	Adequacy of follow-up	
Zhou, 2024 (29)	★	★	★	-	★	-	-	★	5
Zhu, 2024 (30)	★	★	★	-	★	-	-	★	5
Kojima, 2023 (31)	★	★	★	★	★	-	-	★	6
Liu Y, 2021 (32)	★	★	★	-	-	-	-	★	4
Heiland, 2019 (33)	★	★	★	★	★	-	-	★	6
Jonkman, 2018 (34)	★	★	★	-	★	-	-	★	5
Storeng, 2018 (35)	★	★	★	-	★	-	-	★	5
Artaud, 2013 (36)	★	★	★	★	★	★	-	-	6
Alexandre, 2012 (37)	★	★	★	★	★	-	-	-	5
Feart, 2011 (38)	★	★	★	-	★	-	-	★	5
Balzi, 2010 (39)	★	★	★	-	★	★	-	-	5
Wang, 2002 (40)	★	★	★	-	★	★	-	★	6
Wu, 1999 (41)	★	★	★	★	★	-	-	★	6

★Indicates that a star (point) was awarded. - Denotes that no star (point) was awarded. Median cut-off values to discriminate high and low study quality were defined as ≥ and < 5 out of 9 points, respectively, for longitudinal studies.

### 3.3 Predictive value of daily health behavior

The included studies delved into a wide array of daily health behaviors and their implications for predicting ADL disability in older adults. These behaviors encompassed exercise (29, 30, 32, 34, 36, 39–41), physical inactivity (33, 35), sedentary lifestyle (35, 37), smoking (36, 40), alcohol consumption (34, 40), soy product intake (31), sleep duration (35), fruit and vegetable consumption (36), adherence to a Mediterranean-type diet (38), and energy intake (39). We systematically assessed the evidence for the predictive capacity of each health behavior indicator on ADL disability, as detailed in Table 4.

**Table 4:** Predictive strength of daily health behavior indicators for ADL disability.

Daily health behavior indicator	Total number of studies	Number of studies, only including participants free of disability at baseline, that reported a significant increased risk of ADL disability (++)	Number of studies, including both participants free and not free of ADL disability at baseline, that reported a significant increased risk of ADL disability (+)	Number of studies reporting no significant increased risk of ADL disability (-)
Exercise	8	1 (36)	-	7 (29, 30, 32, 34, 39–41)
Physical inactivity	2	1 (33)	1 (35)	-
Sedentary lifestyle	2	1 (37)	1 (35)	-
Smoking	2	1 (36)	1 (40)	-
Alcohol use	2	-	1 (34)	2 (34, 40)
Sleeping	1	-	1 (35)	-
Soy product intake	1	-	1 (31)	-
Consuming fruit and vegetables	1	1 (36)	-	-
Mediterranean-type diet	1	-	-	1 (38)
Higher energy intake	1	-	1 (39)	-
Alcohol use	1	-	-	1 (40)

#### 3.3.1 Exercise

Eight studies have provided information regarding the predictive value of exercise for ADL disability among older adults. These eight studies were conducted on separate cohorts; three studies exclusively examined older adults initially without disability (34, 36, 41), while the remaining studies considered cohorts with mixed disability status at baseline (29, 30, 32, 39, 40). Seven studies concluded that older adults who report daily exercise have a significantly lower risk of developing ADL disability (29, 30, 32, 34, 39–41). Artaud’s analysis showed that, compared to high physical activity, low/intermediate physical activity (hazard ratio 1.72, 95% confidence interval 1.48 to 2.00) was independently associated with an increased hazard of disability (36).

#### 3.3.2 Physical inactivity

Both studies examining the impact of physical inactivity-one with participants initially free of disability (33) and the other with a mixed cohort (35) found a heightened risk of ADL disability among inactive older adults.

#### 3.3.3 Sedentary lifestyle

Evidence from two separate cohorts pointed to a clear link between a sedentary lifestyle and an increased risk of ADL disability, regardless of initial disability status (35, 37). This highlights the importance of promoting active lifestyles among aging populations.

#### 3.3.4 Smoking

Concordant findings from two distinct cohorts indicated that smoking significantly increases the risk of ADL disability among older adults, irrespective of their initial disability status (36, 40). This underscores the need for targeted smoking cessation initiatives within senior care.

#### 3.3.5 Alcohol use

Regarding alcohol consumption, one study reported that moderate use—defined as participants consuming five or more drinks per year without reporting any alcohol-related problems—was associated with more favorable functional outcomes (40). In Jonkman’s study, latent class growth modeling was applied to identify distinct trajectories of functional decline over a 9-year follow-up period. The analysis revealed three distinct linear trajectories as the best solution for both males and females: “no/little decline,” “intermediate decline,” and “severe decline.” The findings indicated that alcohol consumption increased the risk of disable belonging to the intermediate subgroup but decreased the risk of the severe subgroup (34). These results suggest a complex relationship between alcohol use and ADLs among cohorts with varied baseline disability statuses (34, 40).

#### 3.3.6 Sleeping

One study explored the predictive value of sleep duration for ADL disability including participants with and without disability at baseline (35). Storeng’s study identified short (sleeping 6 h or less) or prolonged sleep (sleeping 10 h or more) durations as a critical lifestyle risk factor for ADL/IADL disability (35).

#### 3.3.7 Soy product intake

A single study addressed the impact of soy product consumption on ADL disability, showing a significant trend where infrequent intake was associated with an increased incidence of disability in basic ADLs. This cohort study included only participants free of disability at baseline (31).

#### 3.3.8 Consuming fruit and vegetables

Only one study reported the predictive value of fruit and vegetable consumption for ADL disability, concluding that less than once-daily consumption was associated with an increased hazard of disability. This study included participants with varying baseline ADL statuses (36).

#### 3.3.9 Mediterranean-type diet

Only one study reported the predictive value of a Mediterranean-type diet for ADL disability, finding that in women, adherence to the diet was inversely associated with the risk of incident disability in basic and instrumental ADLs. Women with the highest adherence had a 50% relative risk reduction in incident disability over time compared to those with the lowest adherence (38).

#### 3.3.10 Energy intake

Only one study reported the predictive value of energy intake for ADL disability, concluding that higher energy intake was a significant risk factor for incidents or worsening ADL disability. This study included participants with and without ADL disability at baseline and average daily intakes of energy (kcal) were estimated using the European Prospective Investigation into Cancer and Nutrition food frequency questionnaire (39).

## 4 Discussion

The measurement of health behaviors and ADL is of great significance in public health, medical research, and clinical practice. This systematic review compiles evidence on the influence of various daily health behaviors on ADLs among individuals aged 60 years and above. Our thorough analysis integrated data from 29,180 older adults across 13 longitudinal studies. The findings suggest that physical inactivity, sedentary behavior, smoking, infrequent soy product consumption, short or prolonged sleep duration, inadequate fruit and vegetable intake, and high energy intake are linked to an increased risk of ADL disability among older adults. On the contrary, daily exercise, adherence to a Mediterranean diet (particularly in women), and moderate alcohol consumption were associated with a reduced risk of ADL disability, indicating their potential as protective factors.

However, the current measurement methods are indeed diverse, which to some extent undermines the comparability and reliability of research results. The inability to pool data due to methodological heterogeneity requires a cautious interpretation of these findings. To address this issue, there is a pressing need for more homogeneous studies in the future. By adopting standardized measurement tools, implementing rigorous training and research quality monitoring, fostering interdisciplinary collaboration, and promoting data sharing, we can achieve greater homogeneity in future studies. This approach will not only enhance the comparability and reliability of research results but also enable a more accurate assessment of the impact of health behaviors and ADL on individual health status and quality of life. Ultimately, it will provide a robust scientific basis for formulating more effective interventions.

The emphasis of the included studies was diverse, with the majority investigating the impact of exercise on ADLs. This focus likely reflects the widely recognized importance of physical activity in aging populations (29, 30, 32, 34, 36, 39–41). Significant methodological diversity existed in how daily health behaviors and ADL disabilities were measured across studies, complicating direct comparisons of predictive power. Despite these variations, the collective findings provide valuable insights. For instance, the consistent association between regular exercise and reduced ADL disability across multiple studies underscores its predictive solid value, Healthcare providers and caregivers can confidently promote an active and healthy lifestyle (42) among community-dwelling individuals, and this review’s findings support recent recommendations (43) for moderate-intensity aerobic exercise. Recognized as a ‘pandemic,’ physical inactivity necessitates immediate and strategic public health responses, with global initiatives aiming to reduce its prevalence by 10% by 2025 (43, 44). In our review, physical inactivity and prolonged sitting time were the most critical lifestyle risk factors for ADL disability among older adults, particularly in developing countries, where the long-term health implications of inactivity may not be fully recognized. Exercise is vital to increasing physical activity and combating this issue.

Cigarette smoking is associated with several disabling chronic conditions, such as heart disease, stroke, cancer, and chronic obstructive pulmonary disease (45, 46), and our review suggests a similar association with ADLs, aligning with other studies (47, 48). Our review supports the notion that moderate alcohol use in older populations may reduce the risk or severity of disability, particularly among those with a history of cerebrovascular disease (40). Another study indicated that alcohol consumption increased the risk of disable belonging to the intermediate subgroup but decreased the risk of the severe subgroup (34). These results suggest a complex relationship between alcohol use and ADLs among cohorts with varied baseline disability statuses, highlighting an area for further research (34, 40).

Healthy lifestyle behaviors influencing ADLs have been linked to fatigue (49, 50), with potential mechanisms including sleep patterns, autonomic nervous system abnormalities, biological complexity, and nutritional status. Our review indicates that diet and sleep patterns are associated with ADLs among older adults. Sedentary behavior can negatively impact sleep quality in seniors, leading to insomnia and nocturnal restlessness, which can affect ADL performance (51). Poor sleep quality can result in mental fatigue, precipitating physical dysfunction and compromising ADL performance in older adults (52, 53). The Mediterranean diet (MeDi) is characterized by low consumption of meat and meat products, minimal red meat intake, and very low or no consumption of processed meats. Butter and whole-fat dairy products are consumed in moderation, with a preference for fermented dairy products, cheese, and yogurt (54). The MeDi is well-established for its positive impact on health, quality of life, and longevity (55). An anti-inflammatory diet, characterized by increased consumption of vegetables, fruits, legumes, nuts, whole grains, olive oil, and fish, and limited red meat, fat, and sugar, can alleviate fatigue and improve quality of life in individuals with chronic diseases such as multiple sclerosis (56). The dietary bioactive molecules in this diet, such as omega-3 fatty acids and polyphenols, may activate metabolic pathways that affect inflammation and immunological processes, reducing fatigue and improving ADL performance (56).

Our study consulted that daily soy product consumption may prevent functional ADL decline. Soy products also contain a variety of nutrients and bioactive substances, depending on their type, such as fermented/nonfermented and microorganisms involved in the fermentation. Several epidemiological studies suggesting the disease-preventive effects of soy isoflavones (57), vitamin K_2_ in natto (58), and melanoidins in miso and soy sauce (59). However, the lack of assessment of protein intake as a variable in the study may limit the full understanding of the factors that influence functional capacity in older adults. Previous study indicated that intake of higher amounts of protein was associated with physical functioning in older females with sarcopenia (60). A systematic review indicated that a protein intake higher than the recommended dietary allowance (RDA) was significantly associated with higher Short Physical Performance Battery (SPPB) scores, faster walking speed, greater lower-limb and isometric handgrip strength, and better balance (61). This could create opportunities for future research investigating not only the frequency of soy consumption, but also the quality and total quantity of protein in the diet, helping to clarify its role in maintaining ADLs.

Inadequate fruit and vegetable intake, and high energy intake are linked to an increased risk of ADL disability among older adults. It is not difficult to find that diet quality is closely related to the ADLs of older adults. A review provides observational evidence to support the benefits of diets of higher quality for physical performance among older adults (62). Assmann et al. (63) analyzed data from 21,407 participants of the NutriNet-Santé study with a median baseline age of 55.6 years and found higher adherence to nutrition recommendations (including both diet and physical activity guidelines), were associated with a higher probability to age healthily. Supplementary analyses revealed that this association may, to a small part, be mediated by weight status (63). Future research could strengthen the exploration of the associations between diet quality (such as meal frequency, types of food, dietary preferences, and dietary diversity) and ADLs in older adults, in order to gain a more comprehensive understanding of the relationship between diet and health.

The follow-up periods in the included studies they were varied, with some studies having relatively long follow-up periods. Six studies had a follow-up of 2 to 5 years, and seven follow-ups were longer than 6 years. Understanding whether indicators predict disability in the short-term, long-term, or both. For instance, physical inactivity predicts the development of ADL disability after both 9 years (33) and 12 years (35), and smoking predicts disability after 3.4 years (40) and 12 years (36). Identifying the ‘short-term’ predictive value of daily health behavior indicators is beneficial for targeting older adults who could benefit from preventive interventions against ADL disability. Initiating preventive measures when ‘short-term predictors’ are present is more advantageous than waiting for disability to develop over a more extended period (53). This review highlights the impact of daily health behaviors on ADLs, emphasizing their role not only in improving physical functioning but also in enhancing health-related quality of life (64, 65), self-efficacy (66), and cost-effectiveness (67).

### 4.1 Strengths and limitations

This systematic review is the inaugural effort to consolidate findings from studies that have estimated the longitudinal nexus between daily health behaviors and ADLs among older adults. An exhaustive search strategy was meticulously implemented, complemented by a diligent review of references within the included studies, ensuring a sweeping and thorough assessment of the research query.

This study, however, has its limitations. Our focus was confined to literature in English and Chinese. While this may set certain boundaries on the scope, our profound acquaintance with the domain bolsters our assurance of the expansiveness and understanding of our search methodology. Secondly, the standards for defining daily health behaviors vary across studies, and a uniform framework for evaluating ADLs is also warranted. This gap of standardization can difficult to compare studies and implement effective interventions in different contexts. To address this issue, there is a pressing need for more homogeneous studies in the future. By adopting standardized measurement tools, implementing rigorous training and research quality monitoring, fostering interdisciplinary collaboration, and promoting data sharing, we can achieve greater homogeneity in future studies. Despite these inconsistencies, the aggregate evidence from the extant data substantiates a robust conclusion that underscores the impact of daily health behaviors on the ADLs of the senior populace.

## 5 Conclusion

The synthesis of longitudinal evidence and theoretical underpinnings confirms with robust clarity that daily health behaviors significantly predict independence among older adults. Participation in daily exercise is identified as the most productive behavior for preventing ADL disability, with adherence to a Mediterranean diet and moderate alcohol consumption also manifesting protective advantages. On the flip side, physical inactivity, a sedentary lifestyle, and smoking are strongly correlated with ADL impairment, followed by factors such as short or prolonged sleep duration, infrequent consumption of fruits and vegetables, reduced frequency of soy product intake, and elevated energy intake. The clinical ramifications of these findings are profound, presenting a roadmap for the preemptive identification and aversion of potential incapacities in older adults. By mitigating the risk factors that have been pinpointed, healthcare providers can aid in lessening the aggregate disease burden and foster an extension of a healthy, self-reliant existence for older adults.

## Data Availability

The raw data supporting the conclusions of this article will be made available by the authors, without undue reservation.
